# Supplementary material for: Water deprivation-induced hypoxia and oxidative stress physiology responses in respiratory organs of the Indian stinging fish in near coastal zones
Source: PeerJ. 2024 Jan 25;12:e16793. doi: 10.7717/peerj.16793 (PMC10822137; doi:10.7717/peerj.16793)
Supplement: Supplemental Information 2 [file peerj-12-16793-s002.docx]

**
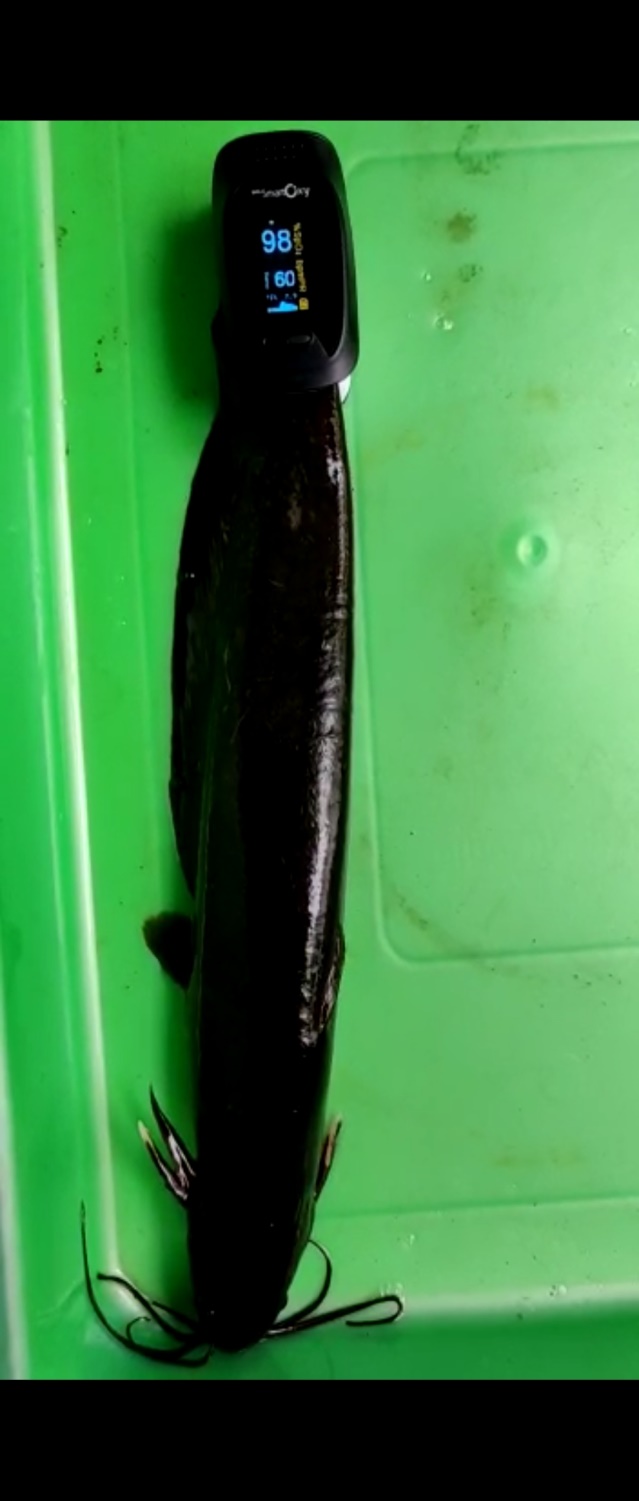
**

**Supplemnetary Fig. 1.** Mesurement of O_2_ saturation level in the fish *H. fossilis* under hypoxia tsress.

A pulse oximeter was used to measure the oxygen stauration in the fish. The readings were recorded at regular intervals of 0, 3, 6, 12 and 18 hours in the respective groups and were computed.
