# Supplementary material for: Water deprivation-induced hypoxia and oxidative stress physiology responses in respiratory organs of the Indian stinging fish in near coastal zones
Source: PeerJ. 2024 Jan 25;12:e16793. doi: 10.7717/peerj.16793 (PMC10822137; doi:10.7717/peerj.16793)
Supplement: Supplemental Information 3 [file peerj-12-16793-s003.docx]

**Supplementary Fig. 2** Correlations graphs of different parameters of ARO tissue with air exposure time period. The level of the oxidative stress parameters (LPx, PC and H_2_O_2_), antioxidant enzymes and non-enzyme molecules (SOD, CAT, GPx, GR, AA and GSH) and respiratory enzymes (Complex I, II, III and V) were correlated with exposure time period.
